# Chromosomal-level reference genome of a wild North American mallard (*Anas platyrhynchos*)

**DOI:** 10.1093/g3journal/jkad171

**Published:** 2023-07-31

**Authors:** Philip Lavretsky, Flor Hernández, Thomas Swale, Jonathon E Mohl

**Affiliations:** Department of Biological Sciences, University of Texas at El Paso, El Paso, TX 79968, USA; Department of Biological Sciences, University of Texas at El Paso, El Paso, TX 79968, USA; Cantata Bio, 100 Enterprise Way Suite A101, Scotts Valley, CA 95066; Department of Mathematical Sciences, University of Texas at El Paso, El Paso, TX 79968, USA

**Keywords:** *Anas platyrhynchos*, wild mallard, chromosome-scale, genome assembly

## Abstract

The mallard (*Anas platyrhynchos*) is one of the most common, economically, and socially important birds around the world. Mallards were not only an important food source for early humans but eventually becoming intimately linked with people as they were domesticated over the last 2,000 years. To date, mallard genomes are largely reconstructed from samples of domestic or unknown genetic heritage. Here, we report the first high-quality genome assembly and annotation of a genetically vetted wild mallard from North America (NAwild_v1.0). The genome was assembled using a combination of shotgun libraries, proximity ligation Chicago, and Dovetail Hi-C libraries. The final assembly is ∼1.04 Gb in size, with 98.3% of the sequence located in 30 full or nearly full chromosome-level scaffolds, and with a N50/L50 of 79.1 Mb/4 scaffolds. We used a combination of gene prediction and similarity approaches to annotate a total of 23,584 functional genes, of which 19,242 were associated to GO terms. The genome assembly and the set of annotated genes yielded a 95.4% completeness score when compared with the BUSCO aves_odb10 dataset. Next, we aligned 3 previously published mallard genomes to ours, and demonstrate how runs of homozygosity and nucleotide diversity are substantially higher and lower, respectively, to ours and how these artificially changed genomes resulted in profoundly different and biased demographic histories. Our wild mallard assembly not only provides a valuable resource to shed light onto genome evolution, speciation, and other adaptive processes, but also helping with identifying functional genes that have been significantly altered during the domestication process.

## Introduction

Even before becoming forever linked with humans as one of the more recently successful domestication events occurring in Eurasia over 2,000 years ago (Larson and Fuller 2014), mallards (*Anas platyrhynchos*) were an important food source dating back to ancient peoples (Jensen *et al.* 2019). This long-standing interlink is exemplified by the myriad of efforts to ensure that mallards thrive in the wild, including their relocation and establishment well outside their native range, as well as increasing the number and types of domestic variants; all of which resulting in these birds having significant socio-economic importance worldwide. In fact, the intentional or accidental release of mallards has increased their range to include the entire world outside the Poles (Baldassarre 2014). Attempts to understand evolutionary mechanisms, consequences of contemporary anthropogenic hybridization, or looking for gene-trait associations to optimize agricultural practices require a contiguous and ancestrally wild reference genome. Although several mallard genomes exist, they are either of specific domestic breeds, un-vetted wild samples (i.e. samples determined to be ancestrally wild prior to full genome sequencing), and/or sequences of pooled individuals. Given the history of captive-reared mallard releases worldwide (Guay and Tracey 2009; Söderquist *et al.* 2017; Lavretsky *et al.* 2020), it is no longer easily assumed that a mallard from the wild is genetically so.

To date, there are 9 mallard genomes published on NCBI (Huang *et al.* 2013; Zhou *et al.* 2018; Liu *et al.* 2020; Li *et al.* 2021; Zhu *et al.* 2021), with all of these being from Eurasian mallards that are either of known domestic origins, and with the 2 “wild” mallard genomes being the result of multiple sample poolings (i.e. CAU-Wild GenBank Accession ID GCA_008746955.1; Zhou *et al.* 2018) and/or of un-vetted origins (i.e. ASM222489V1 GenBank Accession ID GCA_002224895.1; Liu *et al.* 2020; Xi *et al.* 2021). Understandably, genomes have focused on domestic over wild mallards due to their agricultural and medical importance, including understanding gene linkage of favorable agricultural traits (Zhang *et al.* 2018; Zhou *et al.* 2018) and disease resistance in the poultry industry more broadly (Munster *et al.* 2006; Skinner *et al.* 2009). However, these genomes are not appropriate for studies understanding more natural processes or even how wild genomes are transformed through the domestication process due to the constant constraint of artificial selection imposed on domestic lineages (Larson and Fuller 2014). Consequently, we used partial-genome sequences to first establish ancestry of the sample, resulting in the generation of a de novo assembly of a wild mallard collected in New Mexico, United States of America with >98% assignment probability to wild ancestry. In addition, we overlay complementary Topologically Associated Domain (TAD) information, bioinformatically annotate the genome, and demonstrate how the domestication process results in accentuated demographic results that can substantially bias inferences.

## Materials and methods

### Sample collection and DNA extraction

One wild male mallard was collected at Sierra County, New Mexico (32.953 N, −107.295 W). Breast tissue was sent to Dovetail Genomics, LLC (a.k.a Cantata Bio; Scotts Valley, CA) where high molecular weight (HMW) DNA was extracted using the Blood and Cell Culture Midi Kit (Qiagen, GmbH) following the manufacturer's protocol. The bird is curated in the University of Texas at El Paso's Biodiversity Collection (Catalog Number: UTEP-Bird 3056).

### Sequencing and assembly

Extracted DNA was sheared for Illumina library preparation using Bioruptor Pico. Two short-insertlibraries with insert length of approximately 400 and 500 base pair (bp) were prepared (Dovetail Genomics) following the Illumina TruSeq DNA PCR-free protocol. Shotgun libraries were sequenced on an Illumina HiSeq with paired-end 150-bp chemistry. Raw sequences were filtered for sequencing adapters and low-quality bases using Trimmomatic (Bolger *et al.* 2014). Short insert reads were profiled at a variety of k-mer values (19,31,49,75,109), with a negative binomial model fit to k-mer distribution to optimize coverage and achieve a balance between repetitive and heterozygous fractions during assembly. De novo assembly was generated using paired-end libraries with Meraculous v2 (Chapman *et al.* 2011) with a k-mer size of 55 and minimum k-mer frequencies of 15, and the diploid nonredundant haplotig mode.

Using the HMW DNA, 3 Chicago libraries were prepared (Cantata Bio) following the methods described in Putnam *et al.* (2016) In short, ∼500 ng of HMW genomic DNA was reconstituted in vitro into chromatin and fixed with formaldehyde. Fixed chromatin was digested with Dpnll, and with the 5′ overhangs filled in with biotinylated nucleotides, and free blunt ends were then ligated. After ligation, crosslinks were reversed to remove protein from DNA. Purified DNA was treated to remove biotin that was not internal to ligated fragments and sheared to ∼350-bp mean fragment size. Preparation of sequencing libraries was generated from these sheared DNA using NEBNext Ultra enzymes and Illumina-compatible adapters. Biotin-containing fragments were isolated using streptavidin beads before PCR enrichment of each library. Libraries were sequenced for a total of 71 Gb (i.e. ∼70× genomic coverage assuming a ∼1.1-Gbp genome) on a single Illumina HiSeqX lane and using PE 150-bp chemistry.

Next, Omni-C libraries were prepared by Cantata Bio following a modified Hi-C protocol described in Lieberman-Aiden *et al.* (2009). Briefly, chromatin was fixed in place with formaldehyde in the nucleus and then extracted. Fixed chromatin was digested with DNAse I, followed by chromatin end repair and ligation to a biotinylated bridge adapter, and then by proximity ligation of adapter containing ends. After proximity ligation, crosslinks were reversed, and the DNA purified. Purified DNA was treated to remove biotin that was not internal to ligated fragments. Sequencing libraries were generated using NEBNext Ultra enzymes and Illumina-compatible adapters. Biotin-containing fragments were isolated using streptavidin beads before PCR enrichment of each library. The library was sequenced for a total of 68 Gb (i.e. ∼62× genomic coverage assuming a ∼1.1-Gbp genome) on a single Illumina HiSeqX lane and using PE 150-bp chemistry.

The initial de novo assembly from Meraculous, along with shotgun reads, and Chicago library reads were used as input for the HiRise bioinformatics pipeline (Putnam *et al.* 2016). Following, Omni-C library sequences were aligned to the draft input assembly using Burrows Wheeler Aligner v07.15 (bwa; Li and Durbin 2009). Then, the separations of Cantata Bio Omni-C read pairs mapped within draft scaffolds were analyzed by HiRise to produce a likelihood model for genomic distance between read pairs. In addition, the model was used to identify and break putative misjoins to score prospective joins and make join(s) above the default threshold that is automated in the HiRise algorithm. Finally, after aligning and scaffolding the draft assembly using the Chicago data, the Chicago assembly and Omni-C reads were used to improve scaffolding and the mallard assembly using the above method.

Finally, for the wild mallard mitogenome, wild mallard raw Illumina reads were assembled using the mitogenome of the ZJU1.0 pekin duck genome [NCBI BioProject PRJNA20199, version NC_009684.1; (Zhu *et al.* 2021) as a reference in Geneious 10.0.5 (Biomatters Ltd.)]. We ran the internal Geneious mapper with medium sensitivity and up to 5 iterations for fine-tuning. We retained read and base-position with mean quality PHRED Q scores > 30, and positions were genotyped based on a minimum coverage depth of 10 sequences.

### TAD identification

Hi-C contact matrices were generated using the BAM file with read pairs filtered for mapping quality of 60. Topologically Associated Domains (TADs) were identified using Arrowhead within the Juicertool package (Durand *et al.* 2016) with 3 different resolutions, 10 kbp, 25 kbp, and 50 kbp. Clodius was used to create multires files, and HiGlass was used to visualize the results (Kerpedjiev *et al.* 2018). CTCF sites were predicted using the *cread* program (Schones *et al.* 2007) utilizing the position weight matrix from CTCFBSDB 2.0 (Ziebarth *et al.* 2012). Genes were isolated, and significant GO terms with more than expected gene content were identified using in-house python scripts.

### Assembly statistics

Contiguity statistics of scaffolded assembly was computed using Quast (Gurevich *et al.* 2013). BUSCO analysis was performed using BUSCO v3.1.0 (Waterhouse *et al.* 2018) with the associated aves_odb10 dataset. Scaffolds were assigned chromosomal numbers based on alignments to previously published duck genomes (Huang *et al.* 2013; Zhou *et al.* 2018; Li *et al.* 2021; Zhu *et al.* 2021).

### Genome annotation

Chromosomal sequences were passed through Omics-box (https://www.biobam.com) to identify protein-coding regions using Augustus (Hoff and Stanke 2019). Functional annotation of predicted proteins was performed using Omics-Box Functional Genomics package (https://www.biobam.com). In short, a blastp (Altschul *et al.* 1990) search was performed against the nr database, along with InterProScan (Jones *et al.* 2014) run for each of the protein sequences. Within Omics-Box, sequences with significant hits for either the blast or Interpro search were then mapped to a Gene Ontology annotation database (Ashburner *et al.* 2000). The resulting functional annotation table and a wego formatted file with a gene and GO term were exported for use in further analysis. Predicted protein sequences of *Gallus gallus* (chicken, RefSeq: GCF_016699485.2; Warren *et al.* unpublished) and *Anser cygnoides* (goose, RefSeq: GCF_002166845.1; Gao *et al.* 2016) genome assemblies were downloaded from NCBI to compare with the predicted mallard proteins. OrthoVenn2 (Xu *et al.* 2019) was used to cluster the homologous proteins and create a Venn diagram across the different animals. Note that all compared genomes were re-annotated using the same parameters within Omics-box for more direct evaluation.

### Variant calling, genomic diversity, genomic differentiation, and demographic analyses

Variant calling was performed across raw fastq files representing 2 domestic breeds (i.e. CAU-Pekin and CAU-Laying ducks), a mallard of un-vetted wild ancestry (Zhu *et al.* 2021), and our wild mallard using the *process_sequences* script (Python scripts available at https://github.com/jonmohl/PopGen; Lavretsky *et al.* 2020). In short, the process_sequences.py performs the following bioinformatics steps in which poor quality sequences were trimmed and/or discarded with Trimmomatic v0.38 (Bolger *et al.* 2014). Next, quality sequence reads were then aligned to our wild mallard assembly using bwa v07.15 (Li and Durbin 2009). Samples were then sorted and indexed in bcftools v1.14 (Danecek *et al.* 2021) and combined using the bcftools “*mpileup*” function with the following parameters “-c –A -Q 30 -q 30,” which set a base pair and an overall sequence PHRED score of ≥30 to ensure that only high-quality sequences are retained. The resulting VCF file was then filtered using VCFtools v0.1.17 (Danecek *et al.* 2021) with a minimum quality of 30 (-minQ30), a minimum depth of 10 (-minDP 10), and removing all sites with a minimum allele depth of 5 (-remove-filtered ‘AD<5'). Runs of homozygosity and nucleotide diversity was calculated for each genome using VCFtools v0.1.17 (Danecek *et al.* 2021). Whereas all possible base-pairs were used when calculating runs of homozygosity, nucleotide diversity was calculated using a window size of 50 kbp and a sliding window of 5 kbp.

Next, demographic histories were estimated for the same 3 previously published genomes and ours using a Pairwise Sequentially Markovian Coalescent (PSMC) method (Li and Durbin 2011). Genomic data was first filterd for variants having a minimum read sequencing depth of 10, and then followed PSMC parameters optimized for birds as outlined in Nadachowska-Brzyska *et al.* (2015), which included a maximum number of iteration (i.e. *N* = 30), maximum 2*N*_0_ coalescent time (i.e. *t* = 5), initial theta/rho ratio (i.e. *r* = 5), and a pattern of parameters (i.e. p =“4+30*2+4+6+10”). Each analysis was run with 100 bootstrap replicates. Finally, PSMC parameter estimates were converted into biologically informative values based on a generation time (*G*) that was calculated as *G* = *α* + (*s* / (1 − *s*)), where α is the age of maturity and *s* is the expected adult survival rate (Sæther *et al.* 2005). The age of maturity for mallard-like ducks generally is 1 year (i.e. α = 1; Alerstam and Högstedt 1982), and the average survival rate of wild mallards is ~0.57 (i.e. range: 0.46–0.68; Smith and Reynolds 1992; Arnold and Clark 1996; Drilling *et al.* 2020); resulting in an estimated generation time of 2.32 years. The nuclear mutation rate was set to 1 × 10^−9^ (Lavretsky *et al.* 2020).

## Results and discussion

### Genome sequencing and assembly results

We constructed a chromosome-level assembly for a genetically vetted wild mallard (NAwild_v1.0) using a multi-level approach that included 3 different sequencing and assembly technologies (shotgun, Chicago, Omni-C Illumina Seq; Table 1). We generated approximately 510 million paired reads that had a 444.6-fold coverage of the assembly. The assembled genome was ∼ 1.04 Gb with a 40.9% GC content. The scaffold N50 was 79.20 Mb, and the largest contig length was 195 Mb (Table 2). After scaffolding library reads using the Hi-Rise pipeline, the largest 30 scaffolds plus the mitochondrial sequence represented pseudo-chromosomes with a completeness of 95.4% using the aves_odb10 reference. A total of 2,466 unassigned scaffolds added an additional 7 Mb to the overall genome size (Table 2). Together, we saw a reduction in the number of scaffolds with 99.3% of the genome found within the 31 pseudo-chromosomes as compared to previous assemblies (Table 2).

**Table 1.** Sequencing results.

Library type	Bases (Gb)	Coverage (∼1.04-Gb genome)
Shotgun	247.3	237.8
Chicago	153	147.1
Omni-C	62.1	59.7

**Table 2.** Sequencing and assembly results of our wild mallard genome and other publicly available mallard assemblies.

Name	Mallard*^a^*	Mallard*^b^*	BGI_duck_1.0	CAU_duck1.0	ASM222489V1	IASCAAS	ZJU1.0	CAU-Wild	CAU-Pekin	CAU-Laying
Year	2023	2023	2013	2017	2017	2018	2020	2021	2021	2021
Assembly level	Chr. + scaffold	Chr.	Chr.	Scaffold	Scaffold	Chr.	Chr.	Chr.	Chr.	Chr.
Genome coverage	444×	444×	60×	150×	208×	50×	143×	100×	92.6×	93.1×
Total length	1,045,577,032	1,038,030,224	1,105,035,747	1,136,415,614	1,265,073,014	1,126,159,488	1,188,533,289	1,211,992,756	1,186,367,508	1,217,695,176
Largest contig	195,430,658	195,430,658	88,405	52,057,279	17,658,973	202,842,836	207,238,429	208,326,429	207,246,783	212,526,513
# of contigs	2,466	31	227,448	44,791	120,214	73,852	1,661	1,974	618	834
GC%	41.01	40.94	40.5	41.0	41.5	41.5	42.0	42.0	42.0	42.5
N50	79,203,680	79,203,680	1,233,631	74,988,519	2,490,036	76,129,154	76,269,206	77,626,585	76,279,691	76,919,215
L50	4	4	268	5	124	5	5	5	5	5
# N's	5,851,869	5,243,420	35,079,597	56,309,843	95,192,265	2,915,825	4,230,438	1,235,381	1,228,040	7,102,323

Note that waterfowl generally have a consensus diploid (2*N*) number of 80 (Wójcik and Smalec 2007; Basyouny Shahin *et al.* 2014), with autosomal chromosomes 1–29 autosomes and sex chromosomes considered as macro-chromosomes, and the remaining being micro-chromosomes.

Assembly results for our genome.

Assembly results of our genome for the top 31 chromosome-level scaffolds that accounted for 99.3% of the data.

**Fig. 1.**
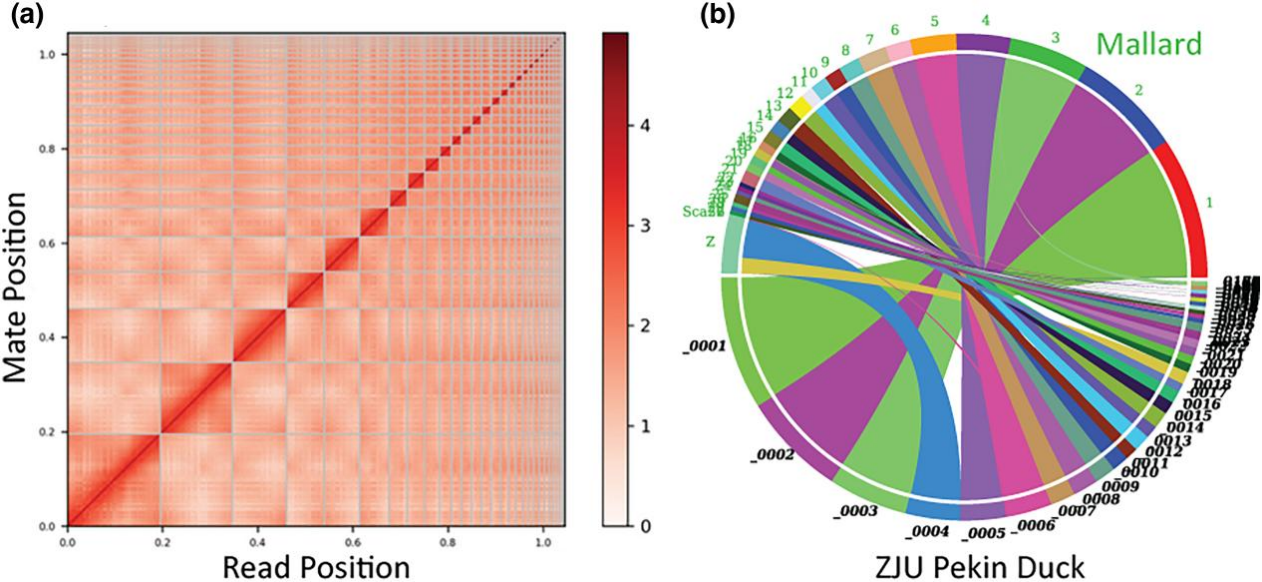
a) Heat map of Omni-C vs HiRise alignment comparisons that show chromosome-level scaffolding, and b) synteny was performed using the Symap program, and against the ZJU Pekin Duck genome.

We acknowledge that long-read technologies (e.g. PacBio) are often superior in resolving repetitive regions that can potentially result in more complete assemblies. However, sequencing Omni-C proximity ligation libraries provided long-range links that still permitted us to join contigs into chromosome-level scaffolds (Fig. 1a). In fact, whereas 37 contigs accounted for 99.5% of our assembled genome, the ZJU genome sequenced with PacBio technology had 98.9% of the assembled genome over 364 contigs (assembly stats are based on contigs ≥50 kb only). This decrease in fragmentation is highlighted by our assembly able to join several smaller fragments in the ZJU genome (e.g. autosomal chromosomes 3 and 17, Z-sex chromosome; Fig. 1b). We conclude that combining Omni-C chemistry and Illumina sequencing attained at least an equivalent assembly as those based on more recent long-read technologies.

### Annotation

Augustus identified 23,584 genes across all the chromosomes and scaffolds. A total of 22,596 (95.8%) predicted proteins were located on the chromosome-scale sequences (i.e. chr1-29 and chrZ). Only 9 of the shorter scaffolds contained 5 or more predicted proteins for a total of 242 and representing 1% of all predicted proteins on those 9 scaffolds. The remaining 2,426 scaffolds had between 0 and 4 predicted genes accounting for the last 746 (3.2%) of all predicted proteins.

**Fig. 2.**
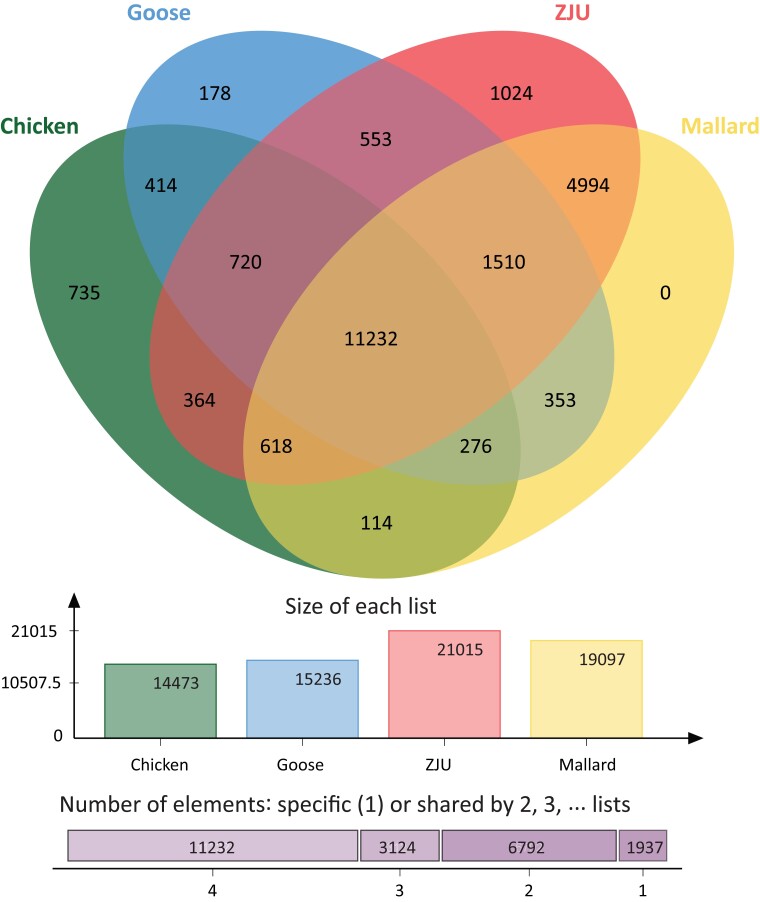
(top) OrthVenn2 Venn diagram highlighting the overlap of the protein clusters of each of the avian species. Singletons (i.e. proteins that did not form a cluster and were not counted within the figure) numbered 11,174 for chicken, 10,380 for goose, 9,153 for ZJU mallard, and 3,888 for our mallard. (middle) Protein cluster counts among the different genomes.The size of the list of proteins in each cluster was balanced suggesting that assembly did not have many duplicated regions. (bottom) Majority of the protein clusters overlapped among all the different taxa (11,232). For the protein clusters between two lists, an increased amount between the ZJU Pekin duck and mallard were found (4,994 of the 6792, or 74%). Furthermore, there was a greater overlap with the 2 ducks and the goose (1,510) then with the chicken (618) as expected based on evolutionary history.

Total predicted proteins found in the genomes was highest in the ZJU mallard (41,531), followed by chicken (33,272 proteins), goose (26,692), and with our wild mallard (23,584) having the lowest number. OrthoVenn2 identified an overlap of 11,232 protein clusters across the 4 predicted sets. From there, protein similarity followed evolutionary histories with greatest recovered overlap in protein clusters with both mallards was with the goose (1,510), vs the chicken (618; Fig. 2). The 2 mallards solely shared an additional 4,994 protein clusters. Interestingly, whereas the ZJU mallard had 1,024 protein clusters unique to it, our wild mallard had no unique clusters. Next, singletons (i.e. proteins that did not form a cluster and were not counted within Fig. 2) numbered 11,174 for chicken, 10,380 for goose, 9,153 for ZJU mallard, and 3,888 for our mallard. In addition to the lack of unique protein clusters within our wild mallard genome, our BUSCO scores were within 1.3% of the other compared genomes, highlighting the similarity and relative completeness of our genome as compared to others. We note that whereas the ZJU mallard genome has less contigs (Table 2), the number of genes identified on non-chromosome-level contigs represented 9.6% (3,988) as compared to 4.2% (1,000) of genes in ours (Fig. 2). The increases in both unique clusters and individual proteins in the ZJU mallard may be due to a more fragmented and/or redundant genome as compared to ours. Alternative to genome completeness, the copy number variation of genes could also be part of the increased number of protein clusters within the ZJU assembly, and which may rather be representative of the type of samples sequenced (i.e. wild vs domesticated strains); the same level of variation is similar to what is even found among people (Gao *et al.* 2023). As more mallards (both wild and agricultural relevant specimens) are sequenced, a better understanding of the variation among populations can be determined.

### TADs

A total of 7,843 potential CTCF binding sites were found using the *cread* (Schones *et al.* 2007) with 2,296 genes found within 12,500 bases to either side of the sites. Only 1 significant GO term was identified, ATP binding (GO:0005524). ATP binding was found to be functionally enriched in the high-altitude adapted populations of buff-throated partridge (*Tetraophasis szechenyii*; Zhou *et al.* 2020), and upregulated during spring migration in black-headed buntings (*Emberiza melanocephala*; Sharma *et al.* 2018). Next, TAD results have been summarized in Table 3. In short, a total of 619 TADs were identified at a resolution of 25 kbp, with a mean size of 592,164 bases that encompassed 33.49% of the genome. The strictest resolution had the lowest number at 558 with a mean size of 235,017 and only 12.58% of the assembly. The 50-kbp resolution had 358 TADs with a mean size of 1,139,106 bases and covered 35.98% of the genome. At the 25-kbp resolution, 6,722 genes were identified within the TADs that are associated with 8 significant GO terms as follows: 4 biological processes (GO:0034765, GO:0006278, GO:0035335, and GO:0050911), 3 molecular functions (GO:0003964, GO:0004725, and GO:0004984), and 1 cellular component (GO:0005634). Of note, genes within the regulation of monoatomic ion transmembrane transport (GO: 0034765) term were shown to be differentially expressed in a feeding study in chickens, which the authors suggest is related to muscle contraction (Juanchich *et al.* 2018). Additionally, genes within the regulation of monoatomic ion transmembrane transport (GO:0034765) were also associated with high altitude adaptions as demarcated through haplotype-based scanning in Rhesus macaques (*Macaca mulatta*; Szpiech *et al.* 2021).

**Table 3.** Topologically Associated Domains (TADs) results called by Arrowhead (Durand *et al.* 2016).

Resolution (kbp)	Number of TADs	Mean TAD size (bp)	Base pair TADs (kbp)	% of genome in TADs
10	558	235,017	1,308	12.58%
25	619	592,164	3,483	33.49%
50	358	1,139,106	3,742	35.98%

### Genetic diversity, runs of homozygosity, and demographic histories

**Fig. 3.**
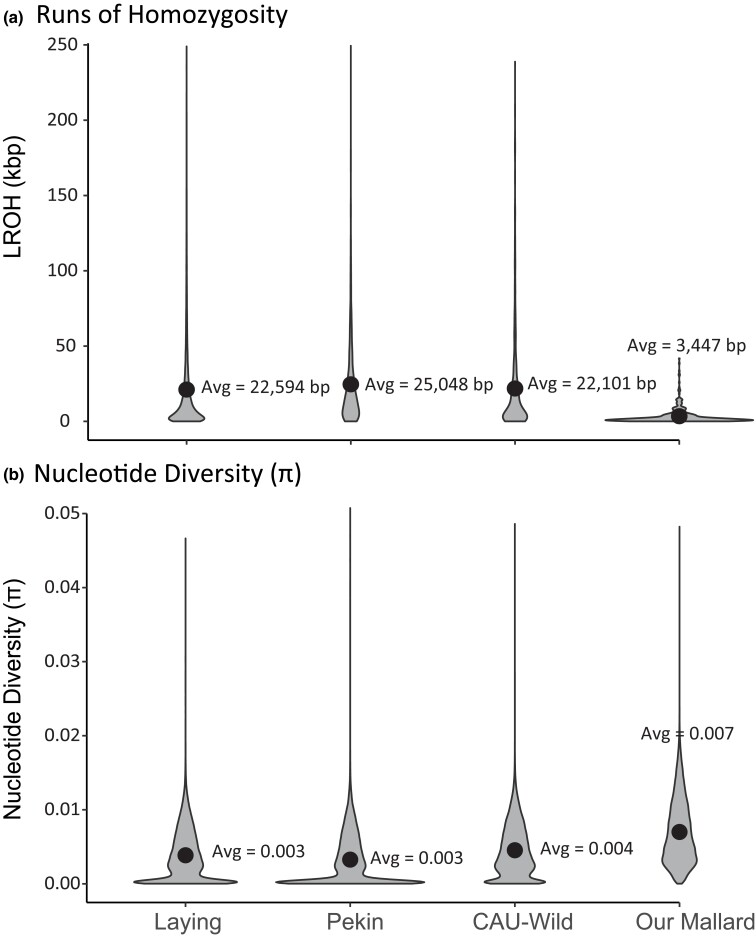
Violin plots of a) long runs of homozygosity (LROH) and b) nucleotide diversity (π) calculated across 2 domestic breeds (CAU-Laying and CAU-Pekin duck), an un-vetted wild mallard (CAU-Wild), and our wild mallard genomes. Note that black dots along with their values denote average LROH and π in each plot.

**Fig. 4.**
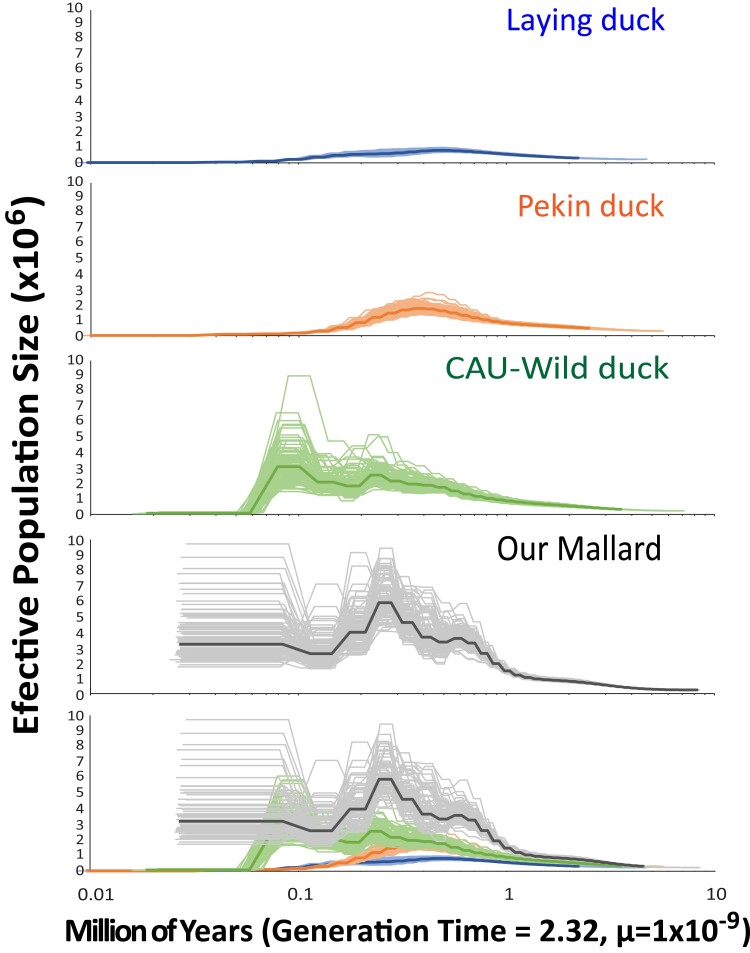
PSMC results for 3 published mallards and our mallard. For each analysis, the dark and lighter lines denote the average and bootstraps, respectively. Deviations in demographic histories suggest a divergence of 1 million years before present, with significant bottlenecking for all but our mallard; showcasing how using domesticated (i.e. CAU-Laying and CAU-Pekin) or unknown origin (i.e. CAU-Wild) breeds can result in substantial deviations in demographic reconstructions, including accentuating divergence times (Brown *et al.* 2022; Lu *et al.* 2022). Most extreme loss in effective population size estimates is among known domesticated breeds, showcasing how sequential bottlenecking with/without artificial selection during the domestication process severely impacts genetic diversity.

A total of 1,030,125,353 quality base pair sites were retained across the 4 genomes: the CAU-Laying, CAU-Pekin, CAU-Wild, and NAwild_v1.0. First, we recovered near similarly low levels of nucleotide diversity (avg. π ∼ 0.003) and long runs of homozygosity (LROH ∼ 23 kbp) for CAU-Laying and CAU-Pekin ducks, followed by the CAU-Wild mallard (i.e. avg. π ∼ 0.004, LROH ∼ 22 kbp), and with our mallard having the highest nucleotide diversity (avg. π ∼ 0.007) and shortest long runs of homozygosity (LROH ∼ 3.5 kbp) (Fig. 3). These calculated parameters suggest that the domesticated ducks have nearly 6.5-times longer runs of homozygosity that translates to 2.4-times less genetic diversity. These differences in genetic diversity translated to substantially differing demographic histories that were recovered across the 4 analyzed mallard genomes, with the greatest exaggerations in effective population size (*N*_E_) and time corresponding with source's respective domestication history, and thus, extent of lost heterozygosity (Figs. 3 and 4). First, the most extreme differences in demographic histories were recovered for the CAU-Laying and CAU-Pekin duck genomes, where both of them show much lower overall peaks of effective population size—CAU-Laying duck *N*_E_ = 81,473 at time 400,000 years before present (YBP) and CAU-Pekin duck *N*_E_ = 168,084 at time 334,650 YBP—after which, both reaching and remaining at near 0 since 45,000 years ago. Next, whereas the general trend between our wild mallard and the CAU-Wild mallard appears similar, they too greatly differ. Specifically, while both genomes show increasing demographic histories starting ∼1 million YBP, the CAU-Wild genome eventually declines to near 0 between 40 and 50,000 YBP, whereas our wild mallard reaches and remains at an effective population size of ∼3 million since 100,000 YBP. Note that the exponential-like increase and contemporary estimates of effective population size are highly concordant with previous estimates using partial genome data for hundreds of vetted wild mallards (Brown *et al.* 2022). Generally, the most extreme loss in effective population size estimates is among known domesticated breeds (i.e. CAU-Laying and CAU-Pekin ducks), showcasing how sequential bottlenecking with/without artificial selection during the domestication process severely impacts genetic diversity (Fig. 3b).

Finally, deviations in demographic histories suggest that the domesticated breeds diverged from their wild ancestor in deep time at nearly ∼1 million YBP. However, animal husbandry and domestication among human civilizations generally occurred between 15,000 and 36,000 years ago with domestication of fowl being one of the last ventures happening over the last 5,000 years (Sossinka 1982; Grayson 2001; Vigne 2011; DeMello 2021). Thus, we argue that inferences made thus far using domesticated (e.g. CAU-Laying and CAU-Pekin) or unknown origin (e.g. CAU-Wild) samples could be misleading, and that our genome provides a less biased demographic history of the species. Together, sample origin is clearly important and caution is required when attempting to infer species’ demographic histories when using genomes of naturally inbred, domestic, or highly admixed individuals (Brown *et al.* 2022; Lu *et al.* 2022).

## Data Availability

The assembled and annotated Wild North American mallard genome (NAwild_v1.0) is available at the National Center for Biotechnology Information (NCBI) Genome Archive under BioProject accession no. PRJNA991977 and sample accession number JAUKTP000000000. All raw sequences associated with NAwild_v1.0 are available from NCBI Sequence Read Archive under BioProject accession no. PRJNA991977 and sample accession number SAMN36329575. Finally, variant calling (VCF) files, and other data used in the analyses are available in UTEP's Bioinformatics Data Repository (https://datarepo.bioinformatics.utep.edu/getdata?acc=JQSHW81NVCY96OE).
